# A Retrospective Single-Center Analysis from Southern Italy on the Use of T2 Magnetic Resonance Assays as a Point-of-Care Method for Patients with Sepsis

**DOI:** 10.3390/biomedicines13040999

**Published:** 2025-04-20

**Authors:** Mariarita Margherita Bona, Vincenza Maria Carelli, Nicola Serra, Salvatore Amico, Roberta Bartolini, Anna Giammanco, Paola Di Carlo, Teresa Fasciana, Maria Andriolo

**Affiliations:** 1 Department of Health Promotion, Mother and Child Care, Internal Medicine and Medical Specialties G. D’Alessandro, University of Palermo, 90127 Palermo, Italy; mariaritamargherita.bona@gmail.com (M.M.B.); anna.giammanco@unipa.it (A.G.); paola.dicarlo@unipa.it (P.D.C.); 2 Clinical Pathology Unit, S. Elia Hospital, 93100 Caltanissetta, Italy; enza.carelli@alice.it (V.M.C.); roberta.bartolini@asp.cl.it (R.B.); andriolomaria@hotmail.com (M.A.); 3 Audiology Unit, Department of Neuroscience, Reproductive Sciences and Dentistry, University of Naples Federico II, Via Pansini 5, 80131 Naples, Italy; nicola.serra@unina.it; 4 Emergency Room Unit, S. Elia Hospital, 93100 Caltanissetta, Italy; salvatore.amico@asp.cl.it

**Keywords:** sepsis, rapid diagnosis, blood culture, PCR, antimicrobial resistance

## Abstract

**Background**: The rapid and accurate identification of the pathogens responsible for sepsis is essential for prompt and effective antimicrobial therapy. The T2Bacteria^®^ Panel (T2B) and T2Candida^®^ Panel (T2C) are rapid molecular tests performed on whole blood that exploit T2 Magnetic Resonance (T2MRsup^®^) technology. **Objectives:** This study evaluates the impact of the T2MR system as a point-of-care device for managing sepsis and septic shock patients. **Methods:** This single-center retrospective study was conducted at the Sant’ Elia Hospital of Caltanissetta from 1 January 2023 to 31 July 2023. The study population was composed of patients with suspected sepsis and septic shock according to the Sepsis-3 criteria and for whom concurrent T2MR and BC samples were requested for diagnosis. **Results:** A total of 81 consecutive patients were enrolled in this study. Concordant T2/BC results were obtained in 69/81 (85.2%) patients; 58/81 (71.6%) were concordant-negative and 11/81 (13.6%) were concordant-positive. Discordant T2MR+/BC− results were observed in 9/81 patients (11.1%), while T2MR−/BC+ results were detected in 3/81 patients (3.7%). Furthermore, the median time for reporting positive T2MR test results (5.2 h) was significantly shorter than that for BC (122 h). **Conclusions:** Due to its high reliability, faster detection time, and simple workflow, T2MR in combination with BC improved the etiological diagnosis of sepsis in the enrolled patients.

## 1. Introduction

According to the Third International Consensus Definitions for Sepsis and Septic Shock (Sepsis-3), sepsis is defined as life-threatening organ dysfunction resulting from a dysregulated host response to infection. Due to its rapid onset, sepsis is considered one of the most challenging and ambiguous medical conditions [1]. It is estimated that bloodstream infections (BSIs) affect approximately 30 million individuals worldwide, leading to 6 million deaths annually [2,3]. Early diagnosis of sepsis is crucial for the timely initiation of effective therapy, which is essential for preventing complications, improving clinical outcomes, and reducing patient mortality. Additionally, prompt diagnosis contributes to the prevention of antimicrobial resistance and limits the spread of multidrug-resistant (MDR) microorganisms, aligning with antimicrobial stewardship strategies [1].

In cases of suspected sepsis, the immediate administration of empirical antibiotic therapy is recommended, as the timing of appropriate definitive treatment is a key determinant of clinical outcomes [4]. Overall, sepsis-related outcomes have significantly improved, likely due to advancements in early diagnostic techniques, the rapid administration of effective antibiotics, and improvements in supportive care for critically ill patients [5]. For these reasons, the rapid and accurate identification of the causative pathogens is fundamental to initiating appropriate antimicrobial therapy. In the absence of a targeted treatment, empirical antibiotic selection is primarily guided by local epidemiological data and resistance patterns [6]. Given the narrow therapeutic window, early and systematic diagnosis of sepsis is critical to preventing the disease progressing to shock, multi-organ failure, or death. Notably, each hour of delay in the administration of an appropriate therapy is associated with a 7.6% decrease in survival rate among sepsis patients [7]. A definitive antibiotic therapy is determined based on the results of molecular diagnostics and/or blood culture (BC) methods, which are considered the reference standard for BSI etiology and the standard of care for sepsis management [8]. However, BC-based methods have the major disadvantage of prolonged turnaround times, typically requiring 24 to 72 h for pathogen identification using automated systems. Additionally, nearly 50% of suspected bloodstream infections remain culture-negative [9].

The implementation of rapid molecular diagnostic assays that enable the early detection of causative pathogens and clinically relevant resistance determinants in the bloodstream of septic patients could significantly reduce the time to appropriate therapy and mitigate antibiotic misuse by facilitating early escalation or de-escalation strategies [10,11,12].

Several molecular diagnostic technologies are currently being evaluated to shorten the time to pathogen identification and, consequently, the turnaround time (TAT) to targeted therapy initiation. T2 Magnetic Resonance (T2MR) technology represents a novel non-culture-based approach for the direct and rapid identification of the most prevalent and clinically relevant bacterial and fungal pathogens from whole-blood specimens. This is achieved through specific diagnostic panels, such as the T2B and T2C panels [13].

The T2Bacteria (T2B) panel, performed on the fully automated T2Dx system (T2 Biosystems, Lexington, MA, USA), enables the identification of six key bacterial species: *Enterococcus faecium*, *Staphylococcus aureus*, *Klebsiella pneumoniae*, *Acinetobacter baumannii*, *Pseudomonas aeruginosa*, and *Escherichia coli*. Collectively referred to as ‘ESKAPE’ pathogens, these organisms are among the most common causative agents of BSIs and frequently exhibit multidrug resistance [14,15,16].

The T2C panel allows for the simultaneous detection of the five most prevalent Candida species in three clinically relevant groups: *Candida albicans*/*tropicalis*, *Candida glabrata*/*krusei*, and *Candida parapsilosis*. According to the U.S. Centers for Disease Control and Prevention (CDC), these five species account for up to 95% of all Candida-related bloodstream infections in the United States [16].

The assay employs a fully automated process integrating polymerase chain reaction (PCR) with probe-enriched superparamagnetic nanoparticles. This enables the detection of superparamagnetic particle agglomerations induced by amplicons, facilitating the identification of bacteria and Candida species within 3–5 h post-blood draw. The technology exhibits high sensitivity, with detection limits ranging from 2 to 11 colony-forming units (CFU)/mL, compared to the >10^7^–10^8^ CFU/mL typically required for BC-based identification [17,18].

The T2B panel has demonstrated high concordance rates with BC-based methods in clinical studies involving patients with suspected BSIs in intensive care units (ICUs), hospital wards, and emergency departments [19,20]. Additionally, the T2 Candida panel has been shown to reduce the time to *Candida* spp. detection with a higher sensitivity than BC, particularly in patients receiving antifungal therapy, where a negative result may effectively rule out active candidemia [21,22,23]. Given these considerations, this single-center retrospective study was designed to evaluate the impact of the T2MR system as a point-of-care diagnostic tool for the management of patients with sepsis or septic shock. This study also aimed to assess the diagnostic accuracy and turnaround time (TAT) of the T2MR assays compared to BC for the detection of bacteremia and candidemia in patients with suspected sepsis or septic shock.

## 2. Materials and Methods

### 2.1. Methods: Study Design

This retrospective observational study was conducted at the Sant’Elia Hospital of Caltanissetta from 1 January 2023 to 31 July 2023. The study population was composed of patients (18 years or older) in whom sepsis and septic shock were suspected according to the Sepsis-3 criteria and for whom concurrent T2 and BC samples were requested for diagnosis [1]. The exclusion criteria included the absence of one of the two tests, samples collected at different times, or insufficient clinical or laboratory data for documentation. Clinical charts were reviewed, and the following data were recorded: age, gender, comorbidities, wards, Sequential Organ Failure Assessment (SOFA), and pathogens detected by T2MR and/or BC. The microbiology laboratory provides diagnostic services to the 420-bed General Hospital of Caltanissetta, Italy, serving a population of approximately 247,118 individuals. The laboratory operates 24 h a day, 7 days a week.

### 2.2. Sample Collection and Processing

To minimize the risk of contamination, blood samples for both T2MR assays and BCs were collected simultaneously under strict aseptic conditions. Three BC bottles were filled with 10 mL of blood for aerobic, anaerobic, and fungal pathogens, while 4 mL K2EDTA Vacutainer blood collection tubes were collected for the T2MR assays.

The blood samples were collected either before admission to the emergency room, during triage, or prior to the administration of antibiotic therapy.

According to clinical routine in Italy, blood samples were collected directly into blood culture bottles using butterfly-type needles or through peripheral vein catheters immediately after insertion. If the patient had a central venous line, one pair of blood cultures were drawn centrally and the other pair were drawn peripherally. All BCs were processed according to standard laboratory protocols [23,24]. Continuous-monitoring blood culture BacT/ALERT^®^ PF Plus (bioMérieux, Durham, NC, USA) was used with a 5-day incubation protocol for bacteria and an 11-day incubation protocol for yeast. All bottles flagged as positive were removed immediately from the instrument, and an aliquot was taken for Gram staining and subculture on agar media, performed automatically on a WCA system. The results from the Gram stains were always reported to clinicians via the phone within 1 h of the start of BC processing. The colony grown on agar plates was identified and its susceptibility was tested by determining minimum inhibitory concentrations to different antimicrobial agents using a VITEK2 system (bioMerieux, Marcy l’Etoile, France), following the standards of the European Committee on Antimicrobial Susceptibility Testing (EUCAST) [24].

For T2MR assays, samples, delivered to the clinical microbiology laboratory during operating times, were processed immediately according to the manufacturer’s instructions. T2MR uses specific panels in order to identify the most prevalent and deadly bacterial and fungal pathogens directly from whole-blood samples. It employs a fully automated process involving PCR and probe-enriched superparamagnetic nanoparticles. Both panels have a low limit of detection (LoD), at 2–11 CFU/mL for the T2B panel and 1–3 CFU/mL for the T2C panel. In addition, it has been demonstrated that T2RM, compared to BC, may shorten the time to microorganism detection with a higher sensitivity, especially during therapy, so a negative result may exclude active bacteremia and/or candidemia [25].

### 2.3. Statistical Analysis

Data are presented as numbers and percentages for categorical variables, and continuous data are expressed as mean ± standard deviation (SD), unless otherwise specified. The inter-rater agreement statistic Cohen’s Kappa unweighted test was used to evaluate the agreement between BC and T2MR tests.

McNemar’s exact test was used to test the differences between paired data. To evaluate the performance of the T2MR test for sepsis evaluation, we used the sensitivity, specificity, and the Receiver Operating Characteristic (ROC) curve, i.e., a graph in which the true-positive rate is plotted as a function of the false-positive rate at different cut-off points, and evaluated the area under the ROC curve (AUC) with standard error (SE) and 95% CI.

To compare two AUCs, the z-test was used. Statistical analysis was performed using the Matrix Laboratory (MATLAB) analytical toolbox version 2008 (MathWorks, Natick, MA, USA) for 32-bit Windows.

## 3. Results

During the study period, 81 consecutive patients with sepsis or septic shock were enrolled. Their median age at testing was 64.2 years, with a standard deviation of 21.6 (63% males (51) and 37% females (30)).

The main characteristics of the study population are summarized in Table 1.

From Table 1, we can observe six patients with two comorbidities. Specifically, one patient had diabetes and solid malignancy, three patients had a history of cardiovascular disease and diabetes, and two patients had a history of cardiovascular disease and solid malignancy.

In Table 2, we evaluate the performance of the T2RM panel to detect patients with sepsis, computing statistical indexes such as sensitivity, specificity, and accuracy, considering BC diagnostic as the gold standard.

**Figure 1:**
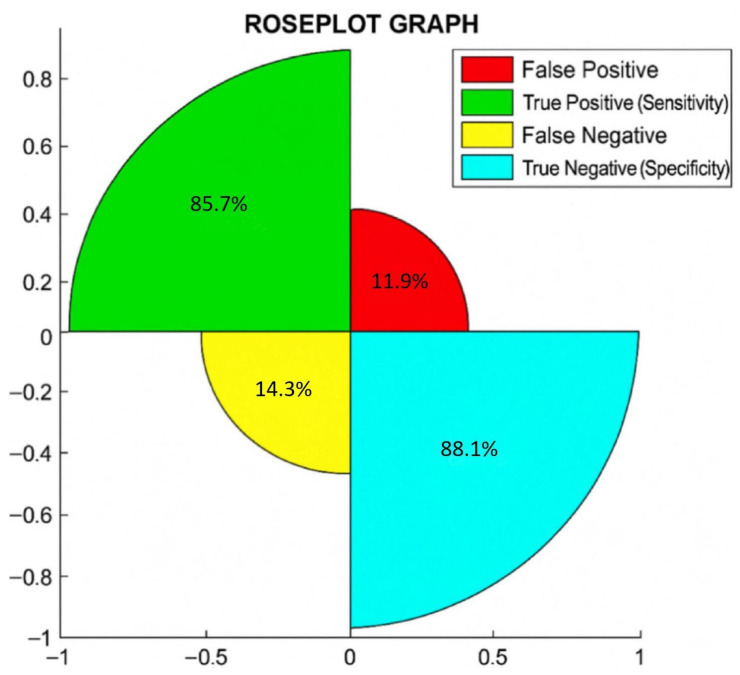
Rose plot shows the percentages of correct and incorrect cases diagnosed by T2RM.

Additionally, in Figure 1, we show the rose plot, where the percentages of true negative or specificity, true positive or sensitivity, false negative, and false positive are reported.

In a rose plot, the area of the segments of the circle conveys amounts, where the angle is constant, i.e., if you divide 360 by the number of parameters considered, this is the square root of the radius that is proportional to percentages.

The rose plot shows that out of 81 patients, 88.1% (59/67 = total negative to BC) were true negative (specificity), 85.7% (12/14 = total positive to BC) were true positive (sensitivity), 14.3% (2/14) were false negative, and 11.9% (8/67) were false positive. Notably, we found a correlation between T2RM and BC in 87.7% (71/81) of cases.

**Figure 2:**
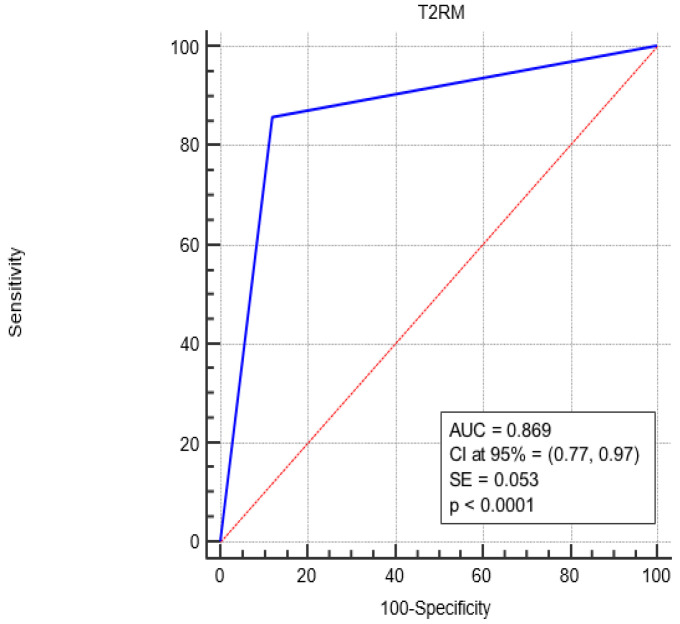
ROC curve analysis for the diagnostic performance of T2RM in sepsis-positive patients. The AUC under the red line is equal to 0.5. Blue Line represents the performance of the T2RM. Red Line represents the diagonal that divide the ROC curve area in two regions. Particularly, all ROC curves with area greater than the diagonal area (0.5) represent diagnostic with good performance while all ROC curves area less than 0.5 represent diagnostic with bad performance.

Figure 2 shows the Receiver Operating Characteristic (ROC) curve analysis, revealing a complete sensitivity/specificity report. The area under the ROC curve (AUC) is reported in the figure compared to an area equal to 0.5.

The AUC associated with T2RM diagnostic performance was significantly greater than 0.5 (0.869 vs. 0.5, *p* < 0.0001), i.e., T2RM is a more accurate diagnostic since the AUC is greater than 0.5.

Finally, we investigated the identified isolated species. Overall, 29 pathogens were identified, 27 of which belong to the species included in the T2RM panel and 2 to other species. The molecular test detected twenty-five of the twenty-six pathogens included in T2B and T2C (one *E. coli*, seven *S. aureus*, five *K. pneumoniae*, one *P. aeruginosa*, one *A. baumannii*, two *E. faecium*, two *C.krusei*/*glabrata*, and two *C. albicans*/*tropicalis*), a number significantly higher (*p* < 0.0001) than that for BC, which detected eleven out of the twenty-seven pathogens (one *E. coli*, one *S. aureus*, six *K. pneumoniae*, two *P. aeruginosa*, and one *E. faecium*). Only one false-negative result was observed for T2RM, while BC failed to identify 14 organisms. Four cases of polymicrobial sepsis were detected only by the T2MR system: one *E. faecium* + *P. aeruginosa* + *C. krusei*/*C. glabrata*, one *K. pneumoniae* + *E. faecium*, one *C. albicans*/*C. tropicalis* + *C. krusei*/*C. glabrata*, and one *C. albicans*/*C. tropicalis*. Yeast was detected by T2MR. Two extra-panel pathogens were isolated by BC: *S. epidermidis* (1) and *S. capitis* (1). The results are reported in Table 3, while the antibiotic resistance profiles of the strains are reported in Supplementary File S1.

Finally, in Table 4, we compare the BC and T2RM diagnostic tools, considering the isolated species and the time needed to perform each diagnostic.

As can be identified in Table 4, we found a significantly good agreement between both diagnostics for blood culture-positive/negative patients considering the isolated species. Notably, in both cases, a significant good association was observed (k = 0.63, *p* < 0.0001 for both).

In addition, the innovative diagnostic was more effective than the conventional method for individualized multiple infections (20% vs. 0.0%, *p* = 0.0153) and for procedure time (median: 5.2 vs. 122, *p* < 0.0001).

## 4. Discussion

Sepsis, a syndrome related to organ dysfunction, is one of the most common causes of increased mortality worldwide. It is the primary cause of death from infection, especially if not recognized and treated promptly. Its detection mandates urgent attention, and rapid and precise identification of the cause of sepsis is crucial for enhancing patient outcomes. Culture-based diagnosis continues to be the gold standard for identifying the causative agent when a bloodstream infection (BSI) is suspected. Unfortunately, the long time required to detect microbial growth, and the possible low load limit this test. PCR systems that provide highly multiplexed targeting of bacterial and/or fungal pathogens (in whole blood) present the greatest potential for clinical impact, as informed decisions can be made within 4–8 h from the blood draw.

Our results show that the use of T2MR testing in combination with the gold standard (BC) method increases the detection rates of the etiological agents involved in sepsis. The principal finding of our study is the high concordance (85.2%) observed between the T2B MRl and BCs for the identification of bacteria in a cohort of critically septic patients, while no correlation (0%) was obtained when T2C and BCs were compared for yeasts. The T2MR system was able to detect positivity in 20 cases (24.7%) compared to 14 positives (17.3%) for BC, and it identified 2 cases of *Candida* spp. which were not detected by BC. It also detected multiple infections, with a 20% higher (four cases) positivity than the culture; in the literature, a positivity of 25% or higher has been reported [13,14,15,16,17,18,19,20,21,22,23,24,25,26].

In addition, the literature highlights that the system allows for a significant reduction in the duration of empiric therapy in patients with negative results for the two diagnostic methods (T2−/BC−). In contrast, in patients with a positive T2MR test, targeted therapy was started based on the test result, especially if the test results were discordant (i.e., T2+/BC−), with a therapy switch rate from empirical to targeted of 66.7%.

In our case, 24.5% of positive cases (20/81) were related to the pathogenic species detected by the T2MR test, as reported in other studies [25]. Considering the microbial species included in the bacteria and mycosis panels, the sensitivity and specificity of the T2MR test were 85.7% and 88.1%, respectively, where BC is considered the gold standard. The high performance of the molecular method (T2MR) is probably due to the high sensitivity of the diagnostic during antimicrobial treatment or may be caused by the occasional presence of microorganisms in the blood when the molecular and cultivation methods are not performed on the same sample.

Moreover, a positive molecular test with a negative culture test could lead to a better prognosis for the patient, as they can be directed toward targeted antibiotic therapy [27,28]. Unfortunately, a false-positive result was detected in the T2MR test for *A. baumannii*.

The T2MR test is not currently approved by the FDA for the detection of *A. baumannii*, which implies that the test’s positivity for this microorganism must be confirmed by requesting and processing a second sample of whole blood.

In this study, we observed that the T2MR diagnostic did not identify one *S. hominis* and one *S. capitis*, in contrast to BC. For BC, this was attributed to the role of contaminants, considering the TTP (131 h for *S. capitis* and 89 h *S. hominis*) and the number of flasks found to be positive. The inability of the T2MR system to identify these microorganisms allows for a reduction in the inappropriate use of antibiotics both when they are considered to be contaminants and when these are involved in serious infections. This represents a limitation of this method and emphasizes that molecular methods should always be used in addition to BC, which, to date, remains the test of choice for sepsis cases [28,29,30,31,32,33,34]. Furthermore, T2B was capable of detecting Gram-negative bacteria that exhibit high levels of antibiotic resistance, such as *A. baumannii*, *P. aeruginosa*, and *K. pneumoniae*. Clinically, this capability may have enabled a significantly faster initiation of an appropriate targeted antibiotic therapy [35,36].

Only in one case was the T2MR test unable to detect the presence of *E. coli*. This result could be related to the different timing of the sampling compared to sample collection for BC, possible contamination of the samples, or mutations in the target site of the amplification [34,35].

The median time for reporting a positive T2MR test result (5.2 h) was significantly less than that for BC (122 h). This was expected because the T2MR test detects the nucleic acid of microorganisms and is not dependent on the growth times of the microorganisms, unlike BC.

In addition, the short time required for sample preparation and initiation of the test allows for a further reduction in the TAT and can easily be applied at night and over public holidays in on-call microbiology laboratories with staff on duty for 12 h shifts, 6 days a week.

Moreover, these results may increase the possibility of including the T2MR system into antimicrobial stewardship programs to reduce the inappropriate use of broad-spectrum antimicrobial drugs [37].

The limited number of microorganisms detected, however, does not allow for the T2MR test to be used without BC, which, in addition to isolating pathogens, is sensitive to antimicrobial drugs. The extreme sensitivity of PCR represents a weakness for the detection of pathogen genes.

In fact, a PCR product can contain millions of amplicons from small amounts of target sequences; this suggests that it detects only low amounts of genetic material derived, for example, from previously contaminated samples by amplification.

Finally, as shown by the ROC curve, a higher AUC value of 0.869 (closer to 1) means that T2RM performs similarly to the BC diagnostic and is effective in detecting both positive and negative cases.

### Limitations

This study has some limitations. It was conducted at a single center and had an observational design and a limited number of enrolled patients. Additionally, information regarding antibiotic therapy adjustments following the T2MR test results was not available. The retrospective nature of this study also introduces a potential collection bias due to the reliance on electronic health records.

Despite these limitations, we believe that the combined use of T2MR and BC can significantly reduce the time to antimicrobial initiation, particularly in patients who have not yet received empirical therapy (due to the faster turnaround time of the T2MR test) or in patients who have to change the therapy on the basis of the identification of microorganisms.

## 5. Conclusions

This pilot study showed the usefulness of potentially using T2RM as a support for BC diagnostics to improve the etiological diagnosis of sepsis and reduce diagnosis times. This could significantly reduce the duration of empiric therapy and enable targeted therapy in patients with positive T2MR test results.

In this group of critically ill septic patients, the T2B panel demonstrated high concordance and was able to identify a greater number of ESKAPE pathogens, with a notably faster turnaround time compared to traditional blood cultures. Additionally, the T2RM has a significant influence on antibiotic and antimycotic treatment decisions. This could be a valuable antimicrobial stewardship strategy, though its impact on clinical outcomes is yet to be determined.

However, pending a study on larger datasets, the results obtained with T2RM can only be considered a support for BC diagnosis.

## Figures and Tables

**Table 1:** Characteristics of the study population.

Parameters	Sample
No. of patients	81
*Age at test*	
Mean ± SD	64.2 ± 21.6
Median (IQR)	71.5 (53.0, 80.0)
*Gender*	
Male	63.0% (51)
Female	37.0% (30)
*SOFA score*	
Mean ± SD	4.9 ± 2.5
Median (IQR)	4.0 (3.0, 6.0)
*Comorbidity* ^†^	54.3% (44)
Chronic liver disease	11.4% (5)
Chronic renal failure	13.6% (6)
Diabetes	15.9% (7)
Hematological disease	2.3% (1)
History of cardiovascular disease	31.8% (14)
Hypertension	15.9% (7)
Solid malignancy	13.6% (6)
*Hospital setting*	
Emergency room	24.7% (20)
Infectious diseases	17.3% (14)
Intensive care	17.3% (14)
Medicine	24.7% (20)
Surgical	3.7% (3)
Other	12.3% (10)
*Leukocytes* (10^3^/µL): Mean ± SD	13.4 ± 12.2
% patients with normal values	39.5% (32)
% patients with abnormal values	60.5% (49)
% *patients with abnormal values*	
Leukocytosis (>11 × 10^3^/µL)	79.6% (39)
Leukopenia (<4.3 × 10^3^/µL)	20.4% (10)
*Platelets* (10^3^/µL): Mean ± SD	226.4 ± 182.0
% patients with normal values	53.1% (43)
% patients with abnormal values	46.9% (38)
*PCR* (mg/L): Mean ± SD	149.38 ± 117.6
% patients with normal values	8.6% (7)
% patients with abnormal values	91.4% (74)
*PCT* (ng/mL): Mean ± SD	17.01 ± 32.88
% patients with normal values	3.7% (3)
% patients with abnormal values	91.4% (78)

SD = standard deviation; IQR = interquartile interval; † = six patients had two comorbidities, Medicine (Internal Medicine, Pulmonology, Nephrology, Hematology, Neurology. Surgical (General Surgical, Vascular Surgical). Others (hemodialysis and gynecology and obstetrics). PCR = C-reactive protein, PCT = Procalcitonin.

**Table 2:** T2RM diagnostic test performance parameters considering BC diagnostic as the gold standard.

Diagnostic	Sensitivity (CI at 95%)	Specificity (CI at 95%)	Accuracy (CI at 95%)
T2MR	85.7% (75.8%, 92.4%)	88.1% (78.5%, 94.2%)	87.2% (78.0%, 93.9%)

**Table 3:** Isolate species identified with BC, T2RM, and both diagnoses.

BC	Both Dignostics	T2MR
*Staphylococcus* spp. (2)	*S. aureus* (5)	*S. aureus* (2)
*E. coli* (1)	*K. pneumoniae* (3)	*K. pneumoniae* (1)
	*E. coli* (3)	*E. coli* (1)
		*A. baumannii* (1)
		*E. faecium* + *P. aeruginosa* + *C. krusei*/*C. glabrata* (1)
		*K. pneumoniae* + *E. faecium* (1)
		*C. albicans*/*C. tropicalis* + *C. krusei*/*C. glabrata* (1)
		*C. albicans*/*C. tropicalis* (1)

**Table 4:** Characteristics of BC and T2RM. In the last column, we report on the comparison between two tests.

Parameters	BC	T2RM	BC vs. T2RM *p*-Value (Test)
*No. of Patients*	81	81	
*Blood culture*			Cohen’s Kappa unweighted = 0.63 (substantial agreement) Standard error = 0.11 CI at 95% = (0.42, 0.84) *p* < 0.0001 *
positive	(14)	(20)	
negative	(67)	(61)	
*Blood culture: characterization* ^#^			
*Bacteria*			Cohen’s Kappa unweighted = 0.63 (substantial agreement) Standard error = 0.066 CI at 95% = (0.50, 0.76) *p* < 0.0001 *
*S. epidermidis*	7.1% (1/14)	0.0% (0)	
*S. capitis*	7.1% (1/14)	0.0% (0)	
*S. aureus*	35.7% (5/14)	35.0% (7/20)	
*K. pneumoniae*	21.4% (3/14)	25.0% (5/20)	
*E. faecium*	0.0% (0)	10.0% (2/20)	
*A. baumannii*	0.0% (0)	5.0% (1/20)	
*E. coli*	28.6% (4/14)	20.0% (4/20)	
*P. aeruginosa*	0.0% (0)	5.0% (1/20)	
*Yeast*			
*C. krusei*/*glabrata*	0.0% (0)	10.0% (2)	
*Candida albicans*/*tropicalis*	0.0% (0)	10.0% (2)	
*Time* (*hours*)			
Mean ± SD	125.6 ± 6.7	5.8 ± 2.1	*p* < 0.0001 * (W)
Median (IQR)	122.0 (113.1, 135.2)	5.2 (4.5, 6.5)	
*Multiple infection*	0.0% (0/14)	20.0% (4/20)	0.0153 * (Mc)

# = In some patients we found a multiple infection; * = significant test; CI = Confidence interval; Mc = McNemar’s exact test; W = Wilcoxon test.

## Data Availability

The original contributions presented in this study are included in the article/Supplementary Material. Further inquiries can be directed to the corresponding author.

## Supplementary Materials

The following supporting information can be downloaded at: https://www.mdpi.com/article/10.3390/biomedicines13040999/s1, File S1: Antibiotics susceptibility profile of isolated bacteria.
